# Evaluating the Translation Process of an Internet-Based Self-Help Intervention for Prevention of Depression: A Cost-Effectiveness Analysis

**DOI:** 10.2196/jmir.2422

**Published:** 2013-01-23

**Authors:** Ove K Lintvedt, Kathleen M Griffiths, Martin Eisemann, Knut Waterloo

**Affiliations:** ^1^Faculty of Health SciencesDepartment of PsychologyUniversity of TromsøTromsøNorway; ^2^Centre for Mental Health ResearchThe Australian National UniversityCanberraAustralia

**Keywords:** Internet, Internet intervention, Cognitive Behavior Therapy, Quality-Adjusted Life Years, Cost Effectiveness, Mental health, Depression

## Abstract

**Background:**

Depression is common and treatable with cognitive behavior therapy (CBT), for example. However, access to this therapy is limited. Internet-based interventions have been found to be effective in reducing symptoms of depression. The International Society for Research on Internet Interventions has highlighted the importance of translating effective Internet programs into multiple languages to enable worldwide dissemination.

**Objective:**

The aim of the current study was to determine if it would be cost effective to translate an existing English-language Internet-based intervention for use in a non-English-speaking country.

**Methods:**

This paper reports an evaluation of a trial in which a research group in Norway translated two English-language Internet-based interventions into Norwegian (MoodGYM and BluePages) that had previously been shown to reduce symptoms of depression. The translation process and estimates of the cost-effectiveness of such a translation process is described. Estimated health effect was found by using quality-adjusted life years (QALY).

**Results:**

Conservative estimates indicate that for every 1000 persons treated, 16 QALYs are gained. The investment is returned 9 times and the cost-effectiveness ratio (CER) is 3432. The costs of the translation project totaled to approximately 27% of the estimated original English-language version development costs.

**Conclusions:**

The economic analysis shows that the cost-effectiveness of the translation project was substantial. Hopefully, these results will encourage others to do similar analyses and report cost-effectiveness data in their research reports.

## Introduction

Computer-aided psychotherapy is a promising way to increase accessibility to evidence-based treatment of many mental disorders, such as mood disorders [1]. Depression is an important global public health issue because of its prevalence [2], that it is associated with a high level of disability and disease burden [3], and its social costs [4]. Depression is also associated with increased risk of physical disorders and early death [5,6].

In a systematic review of computer-aided psychotherapy programs, Marks et al [7] found 97 computer-aided psychotherapy programs described in 175 studies, of which 103 were randomized controlled trials (RCT). These studies discussed screening, effectiveness, efficacy, cost-effectiveness, and the dissemination of computer-aided psychotherapy programs within health services. Cohen’s *d* effect sizes for the identified computer-aided psychotherapy systems ranged from 0.2 (small) to 4.3 (extremely large) [7]. For depression, Marks and colleagues [7] identified 9 programs, of which 3 were Internet-based programs evaluated in RCTs (Overcoming Depression on the Internet [8], MoodGYM [9], and netCBT [10]). In their early reviews of Internet-based RCTs of mental disorders, Griffiths and Christensen [11] and Griffiths et al [12,13] identified another program [14]. Since these reviews, at least 16 new Internet-based intervention programs for depression have been deployed. Several of these have been subjected to research evaluation or are in the test phase: Alles onder controle [15], Color your life [16], Deprexis [17], E-couch [18], HealthSteps for Depression [19], Interapy Depression [20], Living Life to The Full On-line [21], MoodCalmer [22], MoodHelper.org [23], MoodMemos [24], myCompass [25], Project Catch-it [26], This Way Up-Depression Course [27], This Way Up-Mixed Depression and Anxiety Course [28], Xanthis [29], and Youth Mental Health [30]. In addition, more than 20 other programs are still being tested and, thus, no published research evidence of the efficacy of the programs are available.

A recent quantitative meta-analysis on Internet and computerized interventions for adult depression found an overall effect size of *d*=0.41 for computer-aided psychotherapy compared to control [31]. A recent meta-analysis by Andrews and colleagues [32] found an overall effect size for depression of Hedges’ *g*=0.78. Most of the research on Internet-based interventions for depression has focused on treatment rather than prevention, and most studies have employed guided rather than pure self-help strategies. One reason for this focus could be that treatment has more immediate benefits than prevention in which the benefits take longer to emerge [33].

In their meta-analysis, Andersson and Cuijpers [31] reported an average effect size of *d*=0.25 for unguided Internet and computerized interventions. It has been suggested that such interventions have higher attrition rates than those involving therapist support [31,34-36]. To date, we are not aware of any published studies that directly compare attrition in therapist-guided compared to unguided interventions. However, even if attrition is higher in unguided Internet interventions, they have the potential to provide significant public health benefits due to the degree of dissemination [33].

Internet-based self-help has the potential to reach target groups with an unmet need for help. In a recent study, we found that two-thirds of the participants completing a trial of an Internet-unguided cognitive behavioral therapy (CBT) program initially reported an unmet need for help with a psychological problem [37]. Briefly, the Internet intervention was associated with a significant reduction in depressive symptoms compared to the control condition, and thus indicated that the intervention facilitated an effective self-help effort among a group of people who normally would not seek professional help. The importance of reaching and engaging this group is obvious given the evidence that low levels of help-seeking intentions among those with mental health problems are associated with suicidal ideation [38].

There is now sufficient evidence to suggest that Internet interventions can be effective in the prevention and treatment of depression [39,18,40,41]. The International Society for Research on Internet Interventions (ISRII) has highlighted the importance of facilitating the dissemination of Internet applications by providing translation into multiple languages [42]. The World Health Organization (WHO) has also suggested that after establishing their efficacy, Internet-based prevention interventions should be disseminated worldwide [33]. One program that has been subjected to a number of trials and found to be effective over 12 months is the English-language Internet program for depression, MoodGYM [43]. There is also evidence of the efficacy of the English-language website BluePages [44], which provides information on the symptoms of and treatment for depression based on scientific evidence, help resources, and depression and anxiety screening tests. Both programs are available as part of a publicly available, free of charge, e-mental health service delivered by The Australian National University (ANU) and funded by the Australian Government [45]. This paper describes the translation of MoodGYM and BluePages into the Norwegian language by a research group at the University of Tromsø in Norway. Reviews in the field of Internet interventions for mental disorders show that studies are lacking cost-effectiveness data [11,46].

The aim of this study is to evaluate the translation process of an Internet-based self-help intervention up to dissemination and to study cost and cost-effectiveness of the project. It will also consider the feasibility of providing access to Internet-based interventions in the national language versions. Further, we discuss some of the challenges experienced during this project and give recommendations for future developments. Finally, we attempt to demonstrate the use of disease-specific scales to estimate quality of life.

## Method

### Prior Work

#### Intention to Use Internet-Based Self-help

Before translating the Internet-based interventions, a study was undertaken to investigate the need for a Web-based self-help intervention [47]. Nearly 31.9% (115/367) of the respondents reported they felt a need for help, but had not sought help; therefore, they had an unmet need for help. Among these respondents, 91.2% (105/115) reported a positive attitude toward using a service like MoodGYM.

#### The Norwegian Version of MoodGYM and BluePages

In February 2006, Norwegian language versions of BluePages and MoodGYM were made available. The planning of this project started in July 2004 and the project was completed in June 2006. The translation of BluePages was formalized in a licensing deed, specifying ANU as the intellectual property owner and the obligations for the collaborating partners. For MoodGYM, the research collaboration agreement specified a nonexclusive, nontransferable license for the translated version during the period of collaboration and joint ownership of the data emerging from the collaboration. The translation of MoodGYM and BluePages was carried out between October 2005 and January 2006. The Norwegian version of the BluePages website was developed by using a 2-phase process. First, a professional translator prepared a Norwegian version of BluePages. In the second phase, the translation was adjusted by the research group at the University of Tromsø in Norway to ensure that it was culturally and clinically appropriate for Norwegian users. The MoodGYM training program was translated in 4 phases. The research group conducted the first phase of translation. In phase 2, clinical professionals with formal competence in cognitive therapy scrutinized the translation from the first phase and made adjustments to the text when necessary. In phase 3, an expert translator of English compared the Norwegian version of the program with the original Australian version and checked for inconsistencies. In the fourth phase, the research group evaluated all changes and finalized the translation.

#### Validating the Norwegian Version

The Norwegian versions of MoodGYM and BluePages were evaluated in a RCT that compared the effect on depressive symptoms of an unguided Internet-based intervention. The Internet condition consisted of a depression information website and a self-help Web application that delivered automated CBT. The participants in the waiting list condition were free to access formal or informal help as usual. This trial was organized as an unguided quasi-indicated prevention intervention. A total of 163 students (mean age 28.2 years) with elevated psychological distress were enrolled into the trial. The intent-to-treat effect size for depressive symptoms was *d*=0.63 and *d*=0.72 for completers [37]. A total of 61.9% (101/163) of the participants remained in the trial at postintervention, including 53% (43/81) of the Internet intervention participants. With respect to adherence, 33% (27/81) of the Internet intervention participants completed on average 63% (3.1/5 modules) of the MoodGYM program without any support or reminders. Among the completers, 63% (27/43) in the experimental condition and 56% (33/59) in the control condition initially reported an unmet need for help. The logs from MoodGYM showed that the 25% of the 81 participants in the experimental condition who did not return the postintervention questionnaire, on average had used almost half of the modules in MoodGYM (2.4/5 modules, 48%). The trial demonstrated a real-world usage of an unguided Internet-based intervention.

#### Cost Estimates

The total cost estimate for the Norwegian version of MoodGYM and BluePages was estimated from the cost of the translation process and the project costs for the Norwegian evaluation trial.

The translation process was accomplished by using 2 members from the Norwegian research group, 3 students in their final year of clinical psychology training, and external resources (translators, psychiatrists, mailing/printing services). Translation costs for employees and students were calculated based on the estimated time consumed and their salaries. The cost for external resources was based on payments for their services.

The trial costs were incurred over 8 weeks from initial participant contact until the completion of the validation trial. This estimate for total costs was based on all expenditures of the validation trial for BluePages and MoodGYM and included the direct costs of mailing and printing during the trial, and also salary for employees and students. Working hours for developing funding applications were not included in the estimates. Because the aim of this study was to evaluate the translation process up to dissemination, the trial costs are included even though these costs could be regarded as research costs.

The initial cost data were in Norwegian kroner (NOK) and dated back to late 2005 and early 2006. For the purpose of this paper, the cost has been adjusted for inflation based on the Norwegian consumer price index into a 2009 price level [48] at an average annual inflation rate of 2.3%. The development cost from ANU dated back to 2001. We have adjusted the cost for inflation based on the Australian consumer price index into a 2009 price level [49], at an average annual inflation rate of 2.9%. The foreign currencies were then converted into Euro (€) based on real exchange rates from January 1, 2010. These rates form the basis of our calculations, rounded up to the nearest €100. The rationale for using the Australian development cost was in the interest of estimating the development cost if the project had been developed from scratch in Norway, and the Australian development cost provided an estimate of this figure. The cost analyses were based on an operating period of 3 years. After such a timeframe, the Internet-based programs need to be maintained, changes made in design, the technology needs to be updated, and so on. Although most of the investment in the intervention can be maintained, there will be some new costs at this point in time.

### Measures

#### Center for Epidemiologic Studies Depression Scale

The Center for Epidemiologic Studies Depression Scale (CES-D) [50] was used as the outcome measure for depressive symptoms. Each item of the CES-D is scored on a 4-point ordinal scale ranging from 0 to 60, with a total score of 16 or higher considered to be depression. Because this criterion has some limitations [51], it will not be used in this paper. For the completers analyses, we used a stratified scale adapted from Rushton and colleagues [52]: subclinical (0-15), mild/moderate (16-23), and moderate/severe (≥24) depression. The internal consistency of the CES-D scale was alpha = .87.

#### Kessler Psychological Distress Scale

The Kessler Psychological Distress Scale (K10) [53] is a scale of psychological distress developed for use in epidemiological surveys. Scores on the K10 scale range from 10 to 50, with higher scores indicating greater distress. People who score under 20 are likely to be well, whereas those scoring between 20 and 24 have a mild mental disorder, those between 25 and 29 have a moderate mental disorder, and those scoring 30 and over have a severe mental disorder. The internal consistency of the K10 scale was alpha = .79.

#### Quality of Life

There has been a growing interest for applying measures for health-related quality of life (QOL) to evaluate health care services, in general. However, in the field of Internet- and computer-based self-help for mental disorders, reviews find that studies lack cost-effectiveness data [11,46]. This is a disadvantage for the field because proper evaluations among interventions and services are not possible. There are several measures available for this purpose: those specific to groups of diseases, and generic measures applied across disease categories [54]. Some produce a single measure as a health index and others assess a profile of scores in different areas of disease. For independent interventions in which it is essential to use average cost-effectiveness ratios and for mutually exclusive interventions, the use of incremental cost-effectiveness ratios is essential [55]. Interventions in which the costs and effects are not affected by the introduction of other interventions are independent interventions. The cost-effectiveness ratio measures how efficiently the intervention can produce an additional quality-adjusted life year (QALY). In this way, the cost-effectiveness of alternative innovations may be compared. The cost-effectiveness ratio (CER) is calculated as the costs of intervention divided by the health gain (eg, life-years gained). The intervention with the lowest CER is preferred [55].

#### The Rosser Classification of Illness States Scale

**Figure 1:**
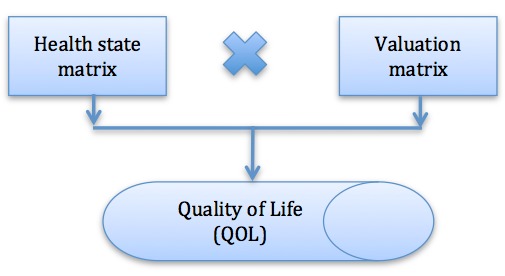
Model for the Rosser Index measuring quality of life (QOL). The health state matrix is based on the Center for Epidemiologic Studies Depression scale (CES-D) scores in relation to the Rosser Disability Category and the Kessler Psychological Distress Scale (K10) scores in relation to the Rosser Distress Category.

The Rosser classification of illness states scale (Rosser Index) [56] offers a ratio scale based on QOL for estimating a utility value that captures the degree of improvement in health (∆H). The ∆H is used to calculate the gain in QALY. As one of the earliest utility instruments, the Rosser Index was developed for use in clinical settings [57]. This is a generic measure with a single index measure for health status. The value for each health state is the sum of all products when multiplying each cell in the Rosser health state matrix with corresponding cells in the Rosser valuation matrix (Figure 1).

In the present study, the Rosser Index was used as a generic instrument to evaluate the observed effects for the Internet intervention. The method is based on classifying outcome data from the trial into 2 components: disability categories (I-VIII) and distress categories (A-D) that define 29 potential health states [56]. The Rosser health state matrix is based on these disability and distress categories.

The original valuation matrix was based on 70 respondents (doctors, nurses, patients, etc) and was not a random sample of the population. The matrix has been transformed and validated in several publications [58]. The Rosser valuation matrix in its present form is based on the method of magnitude estimation [59] in studies from England [56], but they correspond well with Norwegian data [60]. The process of deciding the appropriate categories for the Rosser health state matrix is according to the method described by Gudex and Kind [61].

##### Rosser Health State Matrix: Disability Categories

The CES-D was used as the basis for deciding the appropriate disability categories. No other studies were found that used CES-D as the disability categories in the Rosser health state matrix. The authors have functioned as a reference group of clinical psychologists and found that the CES-D seems to correspond to the Rosser disability dimensions (see Table 1). This is in-line with the recommendations by Gudex and Kind [61]. The first 2 CES-D subgroups (subthreshold and mild/moderate) correspond well with Rosser´s disability categories I (no social disability) and II (slight social disability); the third CES-D subgroup (moderate/severe) corresponds well when dividing the CES-D scores over Rosser´s disability categories III to VI. The disability categories VII to VIII are not used because they represent states of being confined to bed and unconscious. The analogy between CES-D and Rosser Disability category is shown in Table 1.

**Table 1:** Center for Epidemiologic Studies Depression scale (CES-D) scores and their corresponding Rosser disability categories.

CES-D		Rosser disability category	
Subgroup	Score	Category	Description
Subclinical	0-15	I	No social disability
Mild/moderate	16-23	II	Slight social disability, can continue almost as usual with occupational and home activities
Moderate/severe	24-33	III	Severe social disability and/or slight impairment of performance at work
	34-42	IV	Choice of work or performance at work very severely limited
	43-52	V	Unable to undertake any work/education
	53-60	VI	Full-time care or in an institution

##### Rosser Health State Matrix: Distress Categories

The K10 was developed for use in epidemiological surveys and is used to measure psychological distress. No other studies were found that used K10 as the distress category in the Rosser health state matrix. The authors have functioned as a reference group of clinical psychologists and found that the K10 seems to correspond largely with the Rosser distress dimensions (see Table 2). This is in-line with the recommendations by Gudex and Kind [61]. The K10 preassessment scores are used as the basis for deciding the appropriate distress categories for the participants.

**Table 2:** Kessler Psychological Distress Scale (K10) scores and their corresponding Rosser distress categories.

K10		Rosser distress category	
Subgroup	Score	Category	Description
Being well	10-19	A	No distress
Mild mental disorder	20-24	B	Mild distress
Moderate mental disorder	25-29	C	Moderate distress
Severe mental disorder	30-50	D	Severe distress

#### Quality-Adjusted Life Years

A QALY is a year of life adjusted for its quality or its value. A year in perfect health is considered equal to 1 QALY [62]. The value of a year in ill health would be discounted. In the trial, there was a period of 8 weeks between the preassessments and postassessments, in such a way that the ∆H was gained over a 2-month timeframe. The trial did not have a 12-month follow-up, but research from the field shows that the effect over time, from posttest to 12-month follow-up, is maintained [63]. Mackinnon and colleagues [63] conducted a study on 12-month outcome for MoodGYM compared with BluePages and a control group. This gives the best estimate for how to transform our findings to a 12-month outcome. They reported that both the MoodGYM group and the control group had a decline in CES-D scores from posttest to 12-month follow-up, and the control group had the best benefit in reduced symptoms of depression. These transformations of QOL are according to the principles described by Gudex and Kind [61]. The gain in QALYs (QALY-gain) is based on the utility value for improved health ∆H, multiplied by the time interval (T) over which the improvement occurred (measured in years): QALY-gain = ∆H × T [64].

#### Cost per QALY Gained

The National Institute for Health and Clinical Excellence [65,66] in the United Kingdom considers cost per QALY gained among its criteria for coverage recommendations to the National Health Service. This has been converted into an explicit criterion of £30,000 per QALY to be used as a guideline for these recommendations [67]. In Norway, an amount of NOK 500,000 has been proposed (equivalent to €67,100 in 2009) as a temporary best estimate of a QALY [68].

## Results

The Norwegian real cost totalled to €39,900, and the estimated cost to €56,000, adding up to a total of €95,900. Based on data published by Butler and colleagues [69], the Australian development cost was €479,400 plus an average annual maintenance cost of €44,700. The Norwegian costs correspond to almost 27% of the Australian development costs.

Table 3 summarizes the complete cases analysis for depressive symptoms with change scores and effect size [37]. The between-group difference for the Internet intervention and control groups for the overall complete cases analysis was 7.1 points on the CES-D scale and 7.0 points for the intention-to-treat analysis. The mean scores at baseline for K10 were 33.2 (SD 5.5) for the Internet group and 33.2 (SD 5.0) for the control group. The deterioration in the control group is evident in their total scores and rather high numbers in the CES-D subclinical and moderate/severe subgroups. There is almost no change for the mild/moderate subgroup.

**Table 3:** Completers between-group effect size (Hedges’ *g*) for depression level as measured by the Center for Epidemiologic Studies Depression Scale (CES-D).

CES-D scale and condition		Pretest	Posttest	Contrast^a^	Effect size^b^
		Mean (SD), n	Mean (SD), n	Mean (SD)	*g* (SE)
**Total**					
	Intervention	22.6 (10.9), 43	18.5 (14.0), 43	4.1 (10.4)	0.72 (0.21)
	Control	18.5 (9.6), 59	21.4 (13.0), 59	–3.0 (9.1)	
**Subclinical**					
	Intervention	11.4 (3.6), 14	10.0 (8.6), 22	1.4 (8.4)	0.68 (0.34)
	Control	9.8 (3.4), 25	14.4 (9.7), 25	–4.6 (8.8)	
**Mild/moderate**					
	Intervention	19.0 (2.7), 10	11.0 (9.5), 7	8.0 (9.8)	0.85 (0.42)
	Control	19.2 (2.3), 17	19.5 (10.9), 11	–0.3 (9.2)	
**Moderate/severe**					
	Intervention	32.7 (7.0), 19	28.6 (12.8), 14	4.1 (11.7)	0.67 (0.34)
	Control	30.4 (10.0), 17	32.4 (12.1), 23	–3.2 (9.2)	

^a^ Positive contrast represents better outcome.

^b^ Hedges’ *g* is Hedges’ unbiased effect size; SE is Hedges’ unbiased standard error.

The following analysis is based on the completers data from the effect trial [37]. For both conditions (Internet intervention and control group), participants’ distress and disability category is given. Table 4 presents an overview for condition and time in which each cell in the matrix represents the number of cases for the combination of distress and disability. Each cell in this descriptive matrix is associated with a corresponding weight in the Rosser valuation matrix (Table 5).

**Table 4:** Descriptive matrix with number of participants for each disability and distress state, for condition and test time.

Condition	Disability category	Distress category, n							
		Baseline				Posttest			
		A	B	C	D	A	B	C	D
Internet	I	1	8	5		1	12	8	1
	II		6	3	1		4	2	1
	III		3	4	3		2	1	2
	IV		2		5		1	1	5
	V				2				2
Control	I	1	11	10	3		10	12	3
	II		7	4	6		6	3	2
	III		1	7	6	1	3	1	5
	IV		1				1	5	3
	V		1		1				3
	VI						1		

**Table 5:** Rosser´s utility valuation scores for health conditions.

Disability category	Distress category^a^			
	A	B	C	D
I (no social disability)	1.000	0.995	0.990	0.967
II (slight social disability)	0.990	0.986	0.973	0.932
III (severe social disability)	0.980	0.972	0.956	0.912
IV (severely limited work performance)	0.964	0.956	0.942	0.870
V (unable to work/study)	0.946	0.935	0.900	0.700
VI (total social disability)	0.875	0.845	0.680	0.000

^a^ 1 = healthy; 0 =dead. Table shows only relevant disability categories (for complete table see Kind et al [59]).

When all cells in the descriptive matrix are multiplied with the corresponding weight in the valuation matrix, the sum of all these products gives the total QOL score for condition and time. The total QOL score is then divided by the number of participants in the group, which gives an average QOL gain (∆H) score for the group [61]. The Internet intervention group (n=43) had a total QOL at pretest of 40.82 (mean 0.95), and 41.02 (mean 0.95) at posttest, with a ∆H of 0.005. For the control group (n=59), the total QOL at pretest was 56.86 (mean 0.96) and 55.96 (mean 0.95) at posttest, with an average ∆H of -0.015. The between-groups gain in QOL (∆H) was 0.020.

### Prediction of a 12-Month Outcome

By using the change scores from the Australian 12-month follow-up study of MoodGYM and BluePages [63], we calculated a factor to extrapolate our findings to predict a 12-month outcome. Because the results for the control group in our trial were negative, the factors were calculated from posttest to 12-month follow-up. For the MoodGYM group, the change from posttest (mean 15.9) to 12-month follow-up (mean 14.1) was 1.8 points on the CES-D scale, giving a change factor of 1.127. For the control group, the change from posttest (mean 19.5) to 12-month follow-up (mean 16.4) was 3.1 points on the CES-D scale, giving a change factor of 1.19. As the change for the control group was negative, we multiply with the inverse factor at 0.84 (1/1.19). In this way, the difference between groups will diminish.

The gain in QOL (∆H) for the Internet intervention group was 0.0048, and increased to 0.0054 (0.0048×1.127) after transformation. The negative gain in ∆H for the control group was –0.015 and decreased to –0.013 after transformation. The between-groups ∆H reduced to 0.018. The transformation extrapolates the results to a timeframe of 1 year. This gives a QALY-gain=0.018×1, which equals 0.018 of a QALY.

Table 6 presents cost analyses assuming an operating period of 3 years, as the Internet-based programs need to be maintained after some time. The total cost for the translation project is €55,000 per year, equal to 27% of the cost of the development project in Australia. For the translation project, we added an annual cost for maintenance (€14,000) and a service fee (€9000) to provide a more realistic estimate for real-world use. The Internet-based programs run on a shared server in Australia, resulting in the lower maintenance cost for the translation project.

**Table 6:** Estimated development and translation costs with mean cost (based on 3 years’ operating time).

Project and costs type		Costs per year (€1000)			Mean cost (€1000)
		Year 1	Year 2	Year 3	
**Development cost (Australia)**					
	Development	479.4			159.8
	Maintenance	44.7	44.7	44.7	44.7
	Total	524.1	44.7	44.7	204.5
**Translation cost (Norway)**					
	Development	95.9			32.0
	Maintenance	14.0	14.0	14.0	14.0
	Service fee	9.0	9.0	9.0	9.0
	Total	118.9	23.0	23.0	55.0

Based on data from the validation study, the QALY-gain was found to be 0.018 of a QALY. To obtain one QALY, we need 56 individuals (1/0.018) to use the Internet intervention in the same way as the group of completers did in the trial. This is equal to gaining 1 year of full health for 1 person.

Table 7 shows a sensitivity analysis for cost and savings, divided on a development and translation project. Estimated savings per QALY is varied between the Norwegian estimate and the UK estimate. For each of these scenarios, Table 7 shows potential savings based on the number of people treated annually. The CER is the ratio between cost and QALY. The development cost for the translation project is less than the cost for 1 QALY gained, and for the developments project just over 3 QALY gained, based on the Norwegian estimate for cost per QALY gained. The most conservative estimate indicates that for every 1000 persons treated, 16 QALYs are gained. The investment is returned 9 times and the CER is 3432 (based on the more conservative UK estimate for cost per QALY gained). Consequently, if 20,000 people are treated per year, 355 QALYs are gained. Annual savings for the translation project are estimated to at least €500,000 per 1000 persons treated.

**Table 7:** Estimates for cost and annual savings (based on 3 years’ operating time) for development and translation projects, with cost-effectiveness ratio (CER).

Project estimate		Per QALY		Annual					
		Savings (€1000)	Persons	Development cost (€1000)	Persons treated	Savings per person^a^ (€1000)	Total savings (€1000)	QALYs^b^	CER^c^
**Development**									
	Norwegian	67	56	204	1000	0.99	992	15	13,772
	Norwegian	67	56	204	20,000	1.19	23,725	354	576
	UK	30	56	204	1000	0.33	332	11	18,450
	UK	30	56	204	20,000	0.53	10,510	350	582
**Translation**									
	Norwegian	67	56	55	1000	1.20	1141	17	3228
	Norwegian	67	56	55	20,000	1.20	23.875	356	154
	UK	30	56	55	1000	0.54	481	16	3432
	UK	30	56	55	20,000	0.54	10,659	355	155

^a^ Savings per person = (savings per QALY)/(persons per QALY)–(development cost)/(persons treated).

^b^ QALYs = (total savings)/(savings per QALY).

^c^ CER = (development cost)/QALYs.

### Sensitivity Analysis

Our model for estimating the gain in QOL is based on the Rosser Index. Depending on how well the CES-D and K10 scales were fitted to the disability and distress categories, this could affect the output from the model. A sensitivity analysis was conducted to explore how the operationalizing of the scales could have contributed to variation in the outcome (QOL gain). Three scenarios were investigated: (1) the main analysis based on the score for each individual participant, (2) by using the CES-D subgroup mean, and (3) by using the mean for the conditions. In addition, 2 scenarios were investigated to explore the effect of the deterioration in the control group: (4) if there was no change in the control group, and (5) a positive change in the control group. Scenario 1 is already described previously.

Scenario 2 had only 3 values for each condition and time. In the Internet condition, scores for CES-D and K10 placed the CES-D subgroups as follows at pretest and posttest: subclinical group (disability I/distress B, K10 mean 22.8; disability I/distress B, K10 mean 23.7), the mild/moderate subgroup (disability II/distress B, K10 mean 24.4; disability II/distress C, K10 mean 25.3), and the moderate/severe subgroup (disability III/distress D, K10 mean 30.0; disability III/distress D, K10 mean 30.7). In the control condition, scores for CES-D and K10 placed the CES-D subgroups as follows at pretest and posttest: subclinical group (disability I/distress B, K10 mean 24.5; disability I/distress C, K10 mean 25.4), the mild/moderate subgroup (disability II/distress C, K10 mean 27.5; disability II/distress C, K10 mean 25.1), and the moderate/severe subgroup (disability III/distress C, K10 mean 28.5; disability III/distress C, K10 mean 28.3).

Scenario 3 was placed in the disability category II for both conditions, based on CES-D scores (Table 3). The K10 mean was 33.2 for both conditions. The same participants were present at both pretest and posttest, making the distress category D at both the pretest and posttest. When looking at the changes in CES-D scores, it is reasonable to assume that the Internet condition, with a reduction of 4.1 points, resulted in a relief of distress. The opposite could be applied for the control condition, as they had an increase of 3.0 points. The manual for the Rosser Index offers no rules for this scenario, so it seems reasonable to apply a reduction corresponding to half of the change between distress categories C and D, at 0.02. The same applies for the control condition.

Scenario 4 aimed to explore the effect of the deterioration in the control group. If there were no change in the control group during the trial, the QOL gain for the intervention would be equal to the gain for the Internet intervention group. The QOL gain between groups is 0.006, only one-third of the QOL gain from scenario 1.

Scenario 5 represents a scenario in which the control group had a positive change from baseline to posttest, as one could expect during a time period of 12 months. This scenario uses the pretest assessment for both conditions to calculate a new factor to extrapolate our findings to predict a 12-month outcome. The extrapolating was done directly on the pretest scores for each participant to predict outcome scores at 12 months. The rationale for exploring this is based on the fact that the Norwegian and Australian trials were different, unattended vs minimal contact/attention placebo. The change factors for the Internet intervention and control group were 1.546 and 1.317, respectively. The QOL gain between groups is 0.012, ie, smaller than for scenario 1 (two-thirds), but still notable. An alternative approach for this scenario could be to extrapolate the Norwegian posttest scores and compare to the Australian improvement for the control group. The change factors for the Internet intervention and control group would then be 1.127 (as for scenarios 1-3) with a QOL gain of 0.003. This is only one-sixth of the QOL gain from scenario 1. See Table 8 for the results from the sensitivity analysis.

**Table 8:** Quality of life (QOL) sensitivity analysis within and between conditions.

Scenario	Condition	∆H^a^	∆H_ext_^b^
1. Main analysis			
	Internet QOL gain	0.005	0.006
	Control QOL gain	–0.015	–0.013
	Between-conditions QOL gain	0.020	0.018
2. Subgroup mean			
	Internet QOL gain	0.008	0.010
	Control QOL gain	–0.004	–0.003
	Between-conditions QOL gain	0.012	0.013
3. Condition mean			
	Internet QOL gain	0.020	0.024
	Control QOL gain	–0.020	–0.017
	Between-conditions QOL gain	0.040	0.041
4. Main analysis, unchanged control group			
	Internet QOL gain	0.005	0.006
	Control QOL gain	0.000	0.000
	Between-conditions QOL gain	0.005	0.006
5. Main analysis, gain in control group			
	Internet QOL gain		0.024
	Control QOL gain		0.012
	Between-conditions QOL gain		0.012

^a^ ∆H=QOL gain.

^b^ ∆H_ext_ = QOL gain extrapolated.

## Discussion

Conservative estimates indicate that for every 1000 persons treated, 16 QALYs are gained. The investment is returned 9 times and the CER is 3432. The costs of the translation project totalled to approximately 27% of the estimated original English-language version development costs.

### Principal Results

The scenarios in the sensitivity analyses differ in that they decrease in distribution among the 29 potential health states. Scenario 1 uses up to 15 different states, whereas scenario 2 uses up to 5 different states, and scenario 3 only 1 health state. As the distribution becomes more even, the QOL gain (∆H) between groups decreases, and for scenario 3 there is no gain at all. This is partly an effect of the negative results for the control condition. If the control group is kept stable (no change between pretest and posttest score), the QOL change between groups is equal to the change within the intervention condition. Then there is an opposite effect, in which the QOL gain increases with the decrease in distribution. The result is less sensitivity and gives an inflated result. Between scenarios 1 and 2, this is probably a result of less variance and not necessarily that we had to use judgment, as in scenario 3. The results for the extrapolated QOL gain (∆H_ext_) offer no additional insight because it is just a fixed factor that scales the scores into a 12-month QOL gain.

Scenarios 4 and 5 explore the effect of the deterioration in the control group, as one could expect some improvement in that group over a longer time period. The Australian trial is not comparable to the Norwegian because the experimental and control groups are given different focus (minimal contact vs unattended). As scenario 4 shows, we need 3 times as many persons treated to gain 1 QALY. In scenario 5, we need 1.5 as many persons treated to gain 1 QALY.

As the economic analysis shows, the cost of the translation project is less than 1 gained QALY. In the worst case, it is necessary to treat 46 subjects with symptoms of depression to reach the cost break-even point. The treatment received should, on average, be the same as the average for the completers in the trial. As a translation project, this investment is highly cost-effective as it returns the investment many times. With the Norwegian estimate for gained QALY (savings per QALY), every 1000 persons treated yields 17 QALYs and returns the investment 21 times with a CER at 3228. Based on the UK estimate for gained QALY, every 1000 persons treated gives 16 QALYs, returns the investment 9 times, and the CER is 3432. The cost for the translation projects is less than 1 QALY, so the annual number needed to treat to make the cost break-even is 46 individuals, and the development project needs 171 individuals (based on Norwegian estimate for savings per QALY). This is promising, as we know that people, in general, are positive to Internet-based self-help and that this intervention can serve as a prevention intervention. Results from a meta-analysis show that preventive interventions can reduce the incidence of depressive disorders by 22% [70]. The authors concluded, for people with subthreshold symptoms or with high-risk situations, preventive interventions might be exactly what they need.

The trial costs are included in development costs, and include testing the Web-based interventions’ effect and effectiveness. The WHO suggests that Internet-based prevention interventions should be disseminated after establishing their efficacy [33]. We consider the process of “establishing efficacy” as a part of the project and recommend others to include trial costs as well.

We regard the assumptions underlying the cost-effectiveness scenarios as conservative, but at the same time it depends on the kind of marketing, that the potential users find the interventions acceptable, and can trust the service provider. Internet-based interventions are costly to develop and require long-term research to test and evaluate their efficacy and effectiveness before they can be disseminated en masse. Locating funding sources for dissemination is often difficult [33]. This paper shows the potential economic benefits of translating Internet-based interventions into other languages, and that the cultural and linguistic transformation of tools developed in other countries are feasible. Hopefully, this paper also contributes to more research reporting cost-effectiveness data in the future. Our findings should encourage others to undertake similar translation activities. These could comprise other mental disorders for which evidence-based interventions exist, including social anxiety disorder, panic disorder, posttraumatic stress disorder, substance misuse, and eating disorders [71-75]. For small countries, like Norway, with a limited number of researchers in the e-mental health field, it is considerably more cost-effective to translate and implement existing interventions than to develop new ones from scratch. This should enable the relatively rapid introduction and dissemination of new interventions to a broad spectrum of the target population. In addition, such an approach could facilitate global dissemination to reduce health disparities across countries and cultures [76,33]. Although we find it considerably more cost-effective to translate existing interventions than to develop new ones from scratch, there could be situations in which the latter option becomes necessary or appears as more beneficial, ie, if for our area of interest no proven effective Internet-based intervention is available, the content of the program does not use the treatment we are interested in, we want to have control over the content and development of the intervention, and if there is no willingness from intervention developers to give access to their programs.

Internet-based treatments are more accessible than therapist-delivered treatments and, therefore, have the potential to reach more people with an unmet need [77]. An important further step is to assess the cost-effectiveness of the various Internet-based treatments to allow policy makers and health care providers to compare the benefits of online treatment with other interventions. This paper represents one of the few demonstrating the cost-efficacy of an Internet-based therapeutic intervention to treat or prevent a mental disorder and the only study of the cost-effectiveness of translating an existing program for use in another language, as far as we know.

Although the current study demonstrates that the translation of interventions can be cost-effective, there are other issues that need to be addressed in establishing a translation agreement. Firstly, the licensing deed or contract will need to specify the degree to which the translator can adapt and customize the translated version to local requirements. Secondly, this agreement will need to specify the extent to which the translating site will have the opportunity to implement new functionalities, such as customized printouts. Thirdly, the agreement will need to specify the extent to which the translating site will have an opportunity to influence and contribute to new content for the program. If the content of the application is altered, the application is no longer the same. However, if the application is run by the original center, as was the case for MoodGYM and BluePages, the proliferation of different versions represents a logistical challenge, particularly if programs, such as the BluePages depression information website, requires updating as new information develops, or changes are introduced on the basis of user feedback. Fourthly, the contract should be explicit about the ownership of data collected from the translator’s national users and trial. Finally, special attention should be paid to the timely updating of the translated version after changes in the original one.

### Limitations

There are several limitations of this study. The validation part of this paper is based on mixed model repeated measures (MMRM) to handle missing data, which might have introduced some bias. However, the results used in this report were from the complete case analyses as we needed pretest and posttest for all cases to carry out the estimates for QALYs. The effect of the intention-to-treat sample was somewhat smaller than for the complete cases, and our choice of sample could have strengthened the effect and QOL calculations. The deterioration in the control group is not unusual over a short time [78], but over a longer time period, one would expect improvements. The effectiveness study discusses some plausible reasons for this [37], such as that the posttest was conducted just weeks before university examinations started. The usage of completers analysis could have introduced some bias, as well. The effects were considered in a relatively short time span of 8 weeks and should be regarded as potential effect if one completes the trial. There was no long-term follow-up of this trial. How the cost-effectiveness of Internet-based treatment is affected when a longer period of time is used is unknown to us. If an effect is maintained over a longer period, this will increase the QALY gain and thus increase the QALY and reduce the number needed to treat. This assumption was taken into the calculations based on the 12-month outcome data from other studies with MoodGYM and BluePages [63]. All outcome measures are based on self-reports and include only symptoms of depression because no clinical assessment was done. Self-report measures of depression may yield a high rate of false positive and false negative results. This could have compromised the results, identifying persons as depressed who do not meet the criteria for a clinical diagnosis and failing to identify persons with a depressive disorder. No data on sick leave, disability, or other social benefits were collected. The use of such data would have yielded a more complete and realistic cost-effectiveness estimate. However, these benefits would only have increased the estimated savings and present Internet-based interventions in an even more attractive way. Finally, the Rosser Index is just one way to estimate health gains, and other methods could have produced different results [79]. However, this method has been employed previously, and the Rosser Index has its strengths at a policy level often used on existing datasets to foretell health care demands [57]. A similar estimation for QALY gain was conducted to evaluate cost-effectiveness for a face-to-face group intervention designed to decrease depression [80], but they reported only QOL gain on a group level.

### Conclusions

The economic analysis shows that the cost-effectiveness of the translation project was substantial. This is a natural consequence if the project is proven effective in reducing depressive symptoms and based on unguided self-help. The development cost is fixed and the maintenance cost is minimal. Further, as Internet-based treatments are more accessible than therapist-delivered treatments, when these Web-based interventions are disseminated at a national level, they should reach a significant number of individuals with an unmet need for help. The current findings should encourage others to undertake similar translation activities and facilitate global dissemination of effective programs to reduce health disparities across countries and cultures. When data on societal benefits are taken into account, Internet-based prevention interventions should become even more appealing.

## Abbreviations

ANU: Australian National University
CBT: cognitive behavior therapy
CER: cost-effectiveness ratio
CES-D: Center for Epidemiologic Studies Depression Scale
d: Cohen’s d
g: Hedges’ g
∆H: degree of improvement in health
∆Hext: degree of improvement in health, extrapolated
ISRII: International Society for Research on Internet Interventions
K10: Kessler Psychological Distress Scale
MMRM: mixed model repeated measures
NICE: National Institute for Clinical Excellence
NOK: Norwegian kroner
QALY: quality-adjusted life year
QALY-gain: QALYs gained over a timeframe of 1 year
QOL: quality of life
RCT: randomized controlled trial
WHO: World Health Organization
